# Successive stamen movement in *Saxifraga candelabrum* is responsive to weather and pollinator visits

**DOI:** 10.1186/s40462-024-00483-9

**Published:** 2024-06-08

**Authors:** Yumei Luo, Jiming Xie, Lin Zhu, Can Dai

**Affiliations:** 1 School of Resources and Environmental Science, Hubei University, Wuhan, 430062 China; 2 Hubei Key Laboratory of Regional Development and Environmental Response, Hubei University, Wuhan, 430062 China; 3 Xiangshan School Affiliated to Huaibei Normal University, Huaibei, 235099 China

**Keywords:** Cascading stamen movement, Floral behavior, Pollen limitation, Pollen presentation theory, Pollen production, Saxifragaceae, Sex ratio

## Abstract

**Background:**

Successive stamen movement is a complex plant behavior involving successive uplift of stamens and pollen release, which plays a role in reducing sexual interference, increasing pollen deposition and promoting pollen export. Although reported from several taxa, studies on whether the movement can be influenced by abiotic and biotic factors are scarce.

**Methods:**

In this study, we here for the first time described a pattern of successive stamen movement in *Saxifraga candelabrum* (Saxifragaceae). We then compared the rates of stamen movement in *S. candelabrum* under different weather and varying pollinator visits. Pollen packaging and presentation schedule of *S. candelabrum* were also investigated.

**Results:**

The results showed that the number of stamens bent per day in sunny days was significantly higher than overcast and rain. Flowers that receive more pollinator visits (control treatment) had significantly higher number of stamen movement than those that received fewer (removal treatment) and none (bagging treatment). Throughout the staminate phase of a flower, there was a progressive increase in both pollen quantity of individual stamens and pollen presentation during each day.

**Conclusion:**

Our research demonstrates that successive stamen movement in *S. candelabrum* was accelerated by favorable weather and increased pollinator visits, which may promote pollen export. Moreover, incremental pollen packaging is likely an adaptation to seasonal regularity in variations of sex ratio resulting from protandry.

**Supplementary Information:**

The online version contains supplementary material available at 10.1186/s40462-024-00483-9.

## Background

Movements involving floral parts are usually associated with successful pollination [1, 2]. Certain floral movements seem to take place spontaneously, displaying a programmed pattern independent of environmental perturbations. For example, *Alpinia kwangsiensis* T.L.Wu & S.J.Chen (Zingiberaceae) exhibits distinct stigma positions in the morning and afternoon, likely in order to prevent self-pollination [3]. Similarly, flowers of *Passiflora incarnata* L. (Passifloraceae) actively deflex styles to facilitate outcrossing pollen deposition [4]. Yet, some movements, especially rapid ones, are stimulated response to pollinators (i.e. thigmonasty), such as the touch-sensitive stigmas of *Mimulus aurantiacus* Curtis [5], *Campsis radicans* (L.) Seem. [6], and *Mazus miquelii* Makino [7]. In the case of *Berberis* L. [8], *Mahonia bealei* (Fortune) Carr. [8], and *Desmodium* Desv. [9], visiting pollinators cause mechanical stress, leading to the catapulting of filament and explosive pollen release. Moreover, studies on *Cornus canadensis* L. [10] and *Morus alba* L. [11] indicate that explosive pollen discharge can occur both spontaneously and in response to insect movements, suggesting the influence of external factors on spontaneous movements. However, the extent and precise response of such movements to environmental factors are less known.

Various stamen movements have been documented in Berberidaceae [8], Geraniaceae [12], Caryophyllaceae [13], among many others, and the function of stamen movements is commonly considered to minimize sexual interference and improve outcrossing (occasionally selfing as in [14]). The most complex type of stamen movement is, perhaps, the successive movement, one-by-one or group-by-group, found in Parnassiaceae, Rutaceae, Tropaeolaceae, and Loasaceae [15]. In addition to all Parnassiaceae species [16, 17], stamens of *Lychnis cognata* Maxim. [13] and *Geranium pratense* L. [12] have been found to develop in the center of the flower, then successively go through the process of filament elongation, anther dehiscence, pollen release, and finally bending away from floral center. The stamen movements in Rutaceae [18], Tropaeolaceae [19], and Loasaceae [20, 21] are slightly different, with their stamens initially positioned in the same plane with the petals, therefore involving successive anther uplift. In our field observation, the stamens of *Saxifraga candelabrum* Franch. (Saxifragaceae) exhibits a characteristic successive movement like Rutaceae. However, such a movement has not been reported for Saxifragaceae so far (albeit mentioned by Armbruster et al. [16]), nor the factors that may influence stamen movement.

According to pollen presentation theory (PPT) [22–24], the pattern of pollen packaging and dispensing should result in an optimal amount and schedule of pollen doses, which is probably selected to maximize pollen export. For instance, the pollen grains of *Zingiber densissimum* S. Q. Tong & Y. M. Xia are presented on the anther and the labellum staminode simultaneously in the form of aggregated pollen chains, thus promoting pollen output efficiency [25]. Some species of Campanulaceae used pollen-collecting hairs to regulate pollen dispensing and resulted in efficient pollen capture, transfer and deposition [26]. Successive stamen movement seems the ideal mechanism according to PPT, by sequentially maturing and moving stamens, plants can stagger pollen presentation over several days and are capable of adjusting pollen dispensing by altering the speed of stamen movement. For example, some plants of Loasaceae could adjust their stamen presentation patterns with pollinator groups to maximize pollen output [20]. So far, all successive stamen movement reported occurs in species with protandry, in which case carpellate-phase flowers constitute an increasing proportion of a population towards later flowering period [13, 18]. Such a pattern in population sex ratio would result in growing competition for pollen deposition [27, 28]. Indeed, it was found that flowers of protandrous *Chamerion angustifolium* (L.) Holub had varying pollen presentation schedules, with increasing rates of anther dehiscence later in the seasons [29]. In species with successive stamen movements, it is therefore worth investigating whether the rate of movement or quantity of pollen presentation changes within flowers and/or across seasons.

In this study, we aim to describe the patterns of stamen movement in *S. candelabrum*, a herb belonging to the Saxifragaceae, typically growing in rocky habitats of alpine regions. Specifically, we ask whether the movement of stamen can be flexibly regulated by weather and intensity of pollinator visits. Moreover, it is unclear if the amount and schedule of pollen presentation in *S. candelabrum* reflect an adaptation to seasonal ovule availability, which is expected to increase in protandrous species.

## Methods

### Study species

**Fig. 1:**
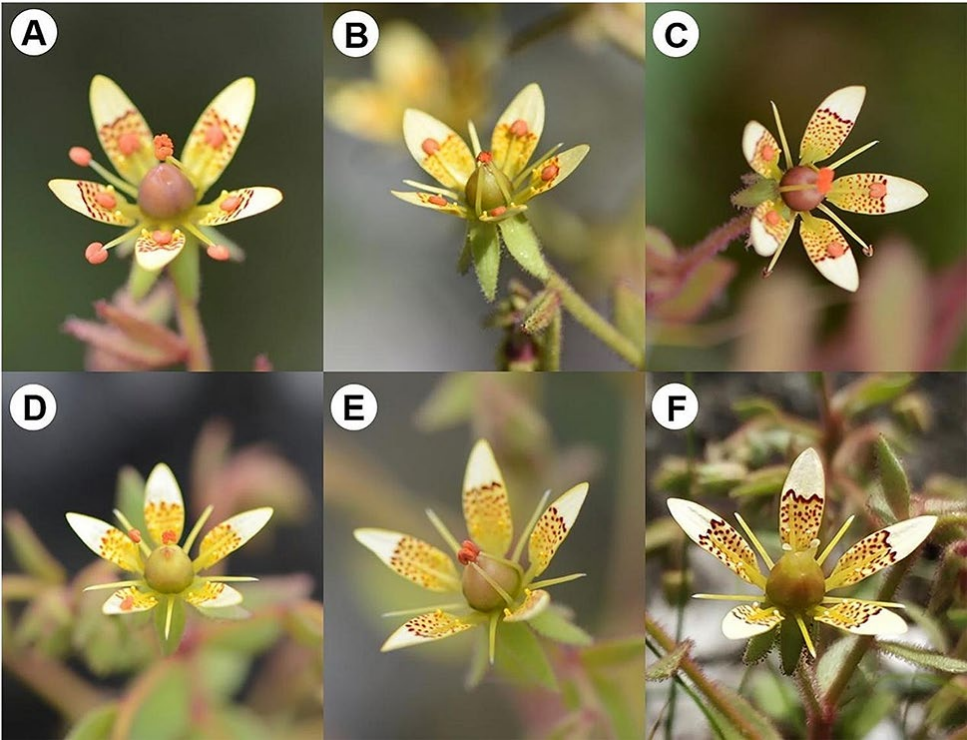
The flowering process of *Saxifraga candelabrum* (**A**-**F**): successive stamen movement in staminate phase (**A**-**E**) and carpellate phase (**F**)

*Saxifraga candelabrum* is a perennial herb widely distributed in alpine meadows of the southwestern of Hengduan Mountains [30]. The plant grows to a height of 19 ~ 38 cm. The inflorescence of *S. candelabrum* is pleiochasium with small light yellow to whitish pentamerous flowers (1.5 ~ 2 cm in diameter). There are purple spots on the petals and nectar secreted at the base of five petals with a peculiar odor. Flowers are strictly protandrous, with the staminate phase (10 stamens, 5 antesepalous and 5 antepetalous [31]) lasting for about 6 days and the carpellate phase 2–3 days (Fig. 1). A neutral phase may occur in-between sexual phases for 1–2 days but it varies much among flowers and plants. Throughout the flowering season, usually from late July to late August or early September, plants of *S. candelabrum* can have up to four or five batches of flowers bloom successively. Around mid-September, the mature capsules release the flattened and dark brown seeds, about 0.5 ~ 1 mm in diameter.

A variety of insects including blow flies, parasitic flies, hoverflies, ants, and beetles visit flowers of *S. candelabrum*. Among them, hoverflies, ants, and beetles only touch petals and feed on nectar, and beetles also consume ovaries. The effective pollinators are probably various kinds of flies (belonging to *Calliphora* and Tachinidae). When they feed on the nectar, their abdomen and legs are in touch with stamens or stigmas of the flowers (Fig. S1).

### Experiment design

The experiments were carried out in two natural populations of *S. candelabrum* at Xia’na (99°02 ‘55 “E, 27°50’ 87” N) and Napahai (99°38 ‘76 “E, 27°54’ 22” N), Shangri-La County, Yunnan Province of China, from July to September, 2019 and 2020. The weather here changed every day, with frequent showers. There were two to three hundred individual plants in each population.

### Reproductive characteristics of *S. candelabrum*

In August 2019 and 2020, we observed the flowering process of nearly two thousand flowers, by visiting daily and recording the status of each flower (staminate or carpellate phase, the number of anthers bending at the moment and bent during the past day). Meanwhile, the quantity of pollen and ovules produced per flower was evaluated. Specifically, one flower in the carpellate phase per plant was taken from 30 randomly chosen plants to quantify ovule number. As for pollen, flowers in the staminate phase were bagged previously to exclude pollinator visits. Since ten stamens in a flower of *S. candelabrum* mature successively (Fig. 1A-E), only the bent stamen (mature) was collected for pollen estimation. The order of bending was also denoted. In total, we harvested 150 anthers from 150 flowers, with equal replications from the first bent stamen till the tenth. Anthers were stored individually in 75% alcohol solution until pollen quantification. Pollen was released and suspended by sonication (KQ-100DB ultrasonic oscillator, Shumei, Kunshan China). The pollen solution was then analyzed by a particle size analyzer (PLD-0203, Puluody, Shaanxi China) and the number of pollen grains in an anther was counted. The order of pollen quantification was randomized among the 150 samples.

To explore the natural pollinator visitation to flowers of *S. candelabrum*, daily observations were conducted from Jul. 30 to Aug. 30, 2020. On each day, we watched pollinator visits on over 30 flowers (with balanced staminate and carpellate phases) in a random 2 m by 2 m square. Each observation period lasted for 15 min between 9:00 and 17:00. The total number of flowers, number of flowers in staminate and carpellate phases, observation period, weather during the observation period, insect species, and duration of each visit were recorded.

We used supplemental outcross pollination to test if the reproduction of *S. candelabrum* suffered from pollen limitation at Napahai in 2020. To avoid biased results due to resource reallocation [32], the experiment was carried out at the plant level, meaning that all blooming flowers of an individual were supplemented with outcross pollen. For each pollinated plant, we selected a plant of similar size and anthesis state nearby as a control, receiving natural pollination. In total, there were 40 groups of supplemental and natural plants.

To make clear if *S. candelabrum* is self-compatible, we conducted self-pollination on 40 randomly chosen plants at Napahai in 2020, one flower per plant. Since the flowers are strictly dichogamous, we employed geitonogamous pollen, directly rubbing the anther sacs onto bifurcated stigmas until saturation. Prior to hand pollination, flowers were kept in bridal-veil nylon bags to ensure a pure pollen source. Bags were removed two days after pollination, in order to allow fruit development under natural conditions. Control flowers were selected randomly from the natural pollination group in the supplemental experiment described above.

By mid-September, when fruits changed color and matured, we recorded fruit set and collected each fruit in a paper bag, air-dried at room temperature. To count the seeds, we spread all the seeds of a single fruit on a piece of paper and used a smart phone (Huawei P30 pro) to take pictures with a fixed focal length of 2 cm. Particle count was performed on digital pictures using Image J (version 1.46, with “size” set to 230).

### Stamen movement and weather effect

In order to understand the full flowering process, especially how stamens move, we made field observations in a natural population of *S. candelabrum* at Xia’na from Jul. 29 to Aug. 17, 2019. From 9:00 to 14:00 every day, we quantified the number of stamens bent over and back in all blooming flowers of randomly selected plants. In total, the flowering process of 1812 flowers from 139 plants was recorded. Meanwhile, the weather at the time of observation was noted, in order to explore the effect of weather (sun, overcast, rain) on stamen movement. We supposed the stamen movement of *S. candelabrum* was not thigmonastic, as no stimulated movement was observed within 10 ~ 30 min after manually touching stamens (both anthers and filaments; as compared to the thigmonastic stamen behavior reported by Schlindwein and Wittmann [33]).

To explore seasonal changes, the flowers in staminate and carpellate phases of all flowering plants in three patches of the same population were quantified every day from Jul. 29 to Aug. 17. The three patches were located in a triangular shape, each about 300 m apart. Moreover, in order to determine whether the rate of stamen movement varies seasonally, we selected two groups of flowers (one random per plant), one categorized as early-season (*n* = 53, blooming from Aug. 1^st^ to 3^rd^) and the other mid-season (*n* = 68, from Aug. 11^th^ to 13^th^). Rates of stamen movement were tallied across the whole staminate phase of each flower. Weather during the two three-day periods were similar, with two sunny days and one overcast day in the early-season and three sunny days in the mid-season.

### Artificial manipulation on flowers and stamen response

To investigate the influence of pollinator visits on stamen movements, we set up three treatments, namely control with open pollination, artificial pollen removal, and bagging with pollinator exclusion. We designed the three treatments to imitate a decreasing gradient of pollinator activity, as the control had pollinators in touch with stamens about 6 times per day (see Fig. S2), whereas artificial removal only had anthers in contact once or twice, and bagging had none. A total of 50 groups of plants were chosen in the Napahai population, each group composed of three plants sharing similar environments with comparable plant sizes and states of anthesis. Three treatments were randomly assigned to plants in a group, and we picked three to four flowers from each plant to conduct experiments. When flowers were in blossom, the open pollination treatment allowed natural pollinators to come and visit without any interference, while the bagging treatment completely excluded pollinators. Artificial pollen removal used small cosmetic soft brushes to rub off pollen in the central mature anther sacs between 10 and 11 am (preliminary experiments demonstrated stamen bending took place between 10 and 11 am). We tried our best to keep the manipulations gentle and precise, without touching other stamens or other floral structures. After 6 pm, when pollinator activity stopped, the state of stamens following the three treatments was recorded (preliminary experiments indicated the stamens did not bend during nights). Before and after the artificial removal, the flowers were kept in bridal-veil nylon bags, just as the bagging treatment. As flowers continued to have new sets of stamens matured and bent, manipulations were repeated every day until the end of staminate phase. The experiments took place during the peak of anthesis from Jul. 30 to Aug. 30, 2020.

### Data analysis

All analysis was done using R version 4.0.2 [34], and packages including car [35], psych [36], lmerTest [37], emmeans [38], lme4 [37], ordinal [39], RVAideMemoire [40] and lattice [41].

To assess whether the reproduction of *S. candelabrum* is self-compatible, t-test and Chi-square test were used to compare seed number and fruiting probability of self- and open-pollinated flowers. To study if the reproduction was pollen-limited, a generalized linear mixed model (GLMM, dist = binomial) was used to compare the fruiting probability of supplemental- and open-pollination, in which the treatment was listed as the fixed effect and group as a random effect. A linear mixed model (LMM) with the same structure was used to analyze the seed number per fruit between supplemental- and open-pollinated plants.

We used ordinal regression with a cumulative link mixed model (CLMM) to explore the effects of weather (sun, rain, overcast) and seasonal changes (early-season vs. mid-season) on stamen movement and the effects of pollinator visits (control, removal, bagging) on the duration of the staminate phase. Since the response variables were integers with limited ranges (number of stamens bent: 0 ~ 4; duration of staminate phase: 4 ~ 8 days), we treated them as ordered categorical data. The fixed factors were weather, season or treatment, respectively. The random factor was flower identity in the weather and season models to control for genotype effects, whereas in the treatment model, group was used as a blocking factor. In the season model, in light of the result that weather affected stamen movement, we also included weather as a random effect. Moreover, because the distributions of both response variables (stamen movement and duration of the staminate phase) were unimodal, we have also checked the data with LMMs assuming normally distributed errors. The statistical results remained qualitatively the same. To investigate how pollinator treatments affect stamen movements (since each flower had multiple entries across its staminate phase), we calculated the average number of stamens bent up per day for all sampled flowers to homogenize the effect of weather. An LMM was then used to test the fixed effect of treatments with group as a random factor.

Quadratic and linear regressions were used to explore how pollen quantity changes with the bending order of stamens and how population sex ratio (carpellate phase/ staminate phase) changes with seasonal progress. Meanwhile, we determined the daily pollen presentation of each flower based on number of stamens bent per day and the corresponding pollen quantity per stamen. Quadratic and linear regressions were also used to explore how pollen presentation changes with the progress of the staminate phase, with weather considered as a random factor. In the above three analyses, adding the quadratic term significantly increased the explanatory power of the regression (quadratic vs. linear: *P* = 0.03 for pollen production; *P* < 0.0001 for daily pollen presentation; *P* < 0.0001 for sex ratio), indicating the nonlinearity of floral and seasonal changes. Therefore, we only reported quadratic regressions in the result. In addition, because the ten stamens are developmentally categorized into two groups, five antesepalous stamens developing first, followed by five antepetalous ones [31]. We used t-test to compare difference in pollen production between the two groups. In light of this ontogenetic effect (see results), we further tested if the bending order of stamens positively influenced pollen quantity within each stamen group using ANCOVA.

In all models with continuous response variables, raw data were used because normality and homogeneity of variance was met. The statistical significance of fixed factors in GLMMs and CLMMs was estimated by model comparisons [42] and LMMs using Satterthwaite’s approximation. Post-hoc comparisons were done with Tukey adjustment in the ‘emmeans’ package. In the result section, we reported least square means ± standard errors unless otherwise specified.

## Results

### Successive stamen movement and reproductive characteristics of *Saxifraga candelabrum*

Our observation of nearly two thousand flowers revealed that flowers of *S. candelabrum* exhibited successive stamen movement. The stamens and petals were in the same plane when the petals were fully open, and ten stamens successively bent to the floral center. The stamens gradually matured and anther sacs dehisced slightly as they bent. When they reached the floral center, anthers were in full dehiscence and pollen release took places, after which they moved back to original positions (Fig. 1A-E). Five antesepalous stamens bent first, followed by the remaining five antepetalous ones (Fig. 1A-E). Within each group of five stamens, there seems no effect of the stamen array on the bending order. The number of bent stamens was not strictly one or two per day, but varied from zero to four anthers day to day, with an average of 1.74 ± 0.88 (mean ± SD), indicating that the dynamics of anther movement are influenced by other factors besides development. Each flower of *S. candelabrum* produced 567.8 ± 62.8 ovules and 173 720 ± 13 356 pollen grains (17 726 ± 5505 pollen per anther, mean ± SD). The P/O ratio was about 306.

Self-pollination on flowers of *S. candelabrum* resulted in 85% fruit-set, while natural pollination led to 87.5% fruit-set, which was of no statistical difference (*Χ*^2^ = 0, *P* = 1). On average, a selfing fruit produced 408.64 ± 30.5 seeds, which was not significantly different from natural seed set (411.23 ± 24.8; *F*_1,61_ = 0.004, *P* = 0.95), indicating full self-compatibility. Supplemental outcross pollination not only increased fruit-set (supplemental: 90.6%; control: 84.5%; *Χ*^2^ = 7.51, *P* = 0.006), but also increased seed number per fruit (supplemental: 418 ± 16.1; control: 362 ± 16.4; *F*_1,486_ = 28.1, *P* < 0.0001). Therefore, fruit reproduction of *S. candelabrum* at the investigated population suffered from pollen limitation.

### Effect of weather on pollinators and stamen movement

**Fig. 2:**
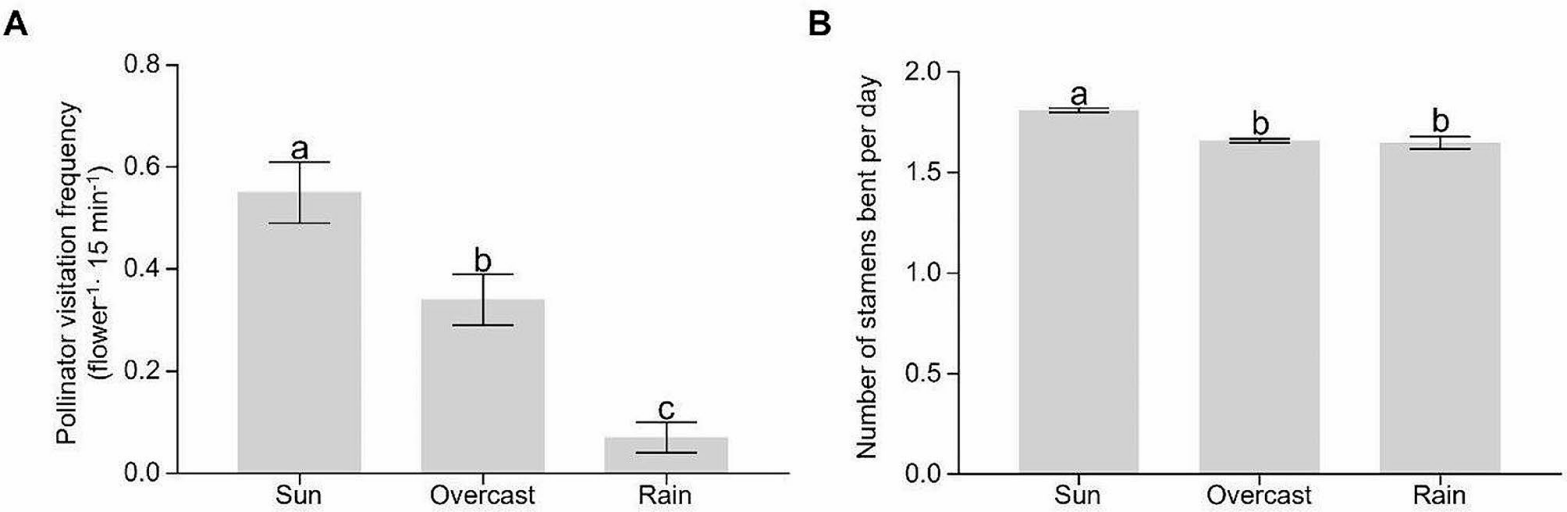
Pollinator visitation frequency on flowers of *Saxifraga candelabrum* (**A**) and number of stamens bent per day (**B**) under different weather conditions

The visiting frequency of pollinators showed a unimodal pattern, peaking around 1300 to 1400 h in sunny and overcast conditions (Fig. S2). Accumulatively, a flower received about 6 pollinator visits per day (Fig. S2). However, the pollinator activity changed with weather conditions (Fig. 2A). The visitation frequency on sunny days was the highest, with about 1.6 times of that on overcast days and 7.8 times of that under rainy conditions (*F*_2,109_ = 9.1, *P* = 0.0002). Correspondingly, the number of stamens bent per day was affected by weather, with significantly more stamens bent up on sunny days than in overcast and rain (*F*_2,9081_ = 32.9, *P* < 0.0001; Fig. 2B).

### Effect of pollinator visits on stamen movements and duration of staminate phase

**Fig. 3:**
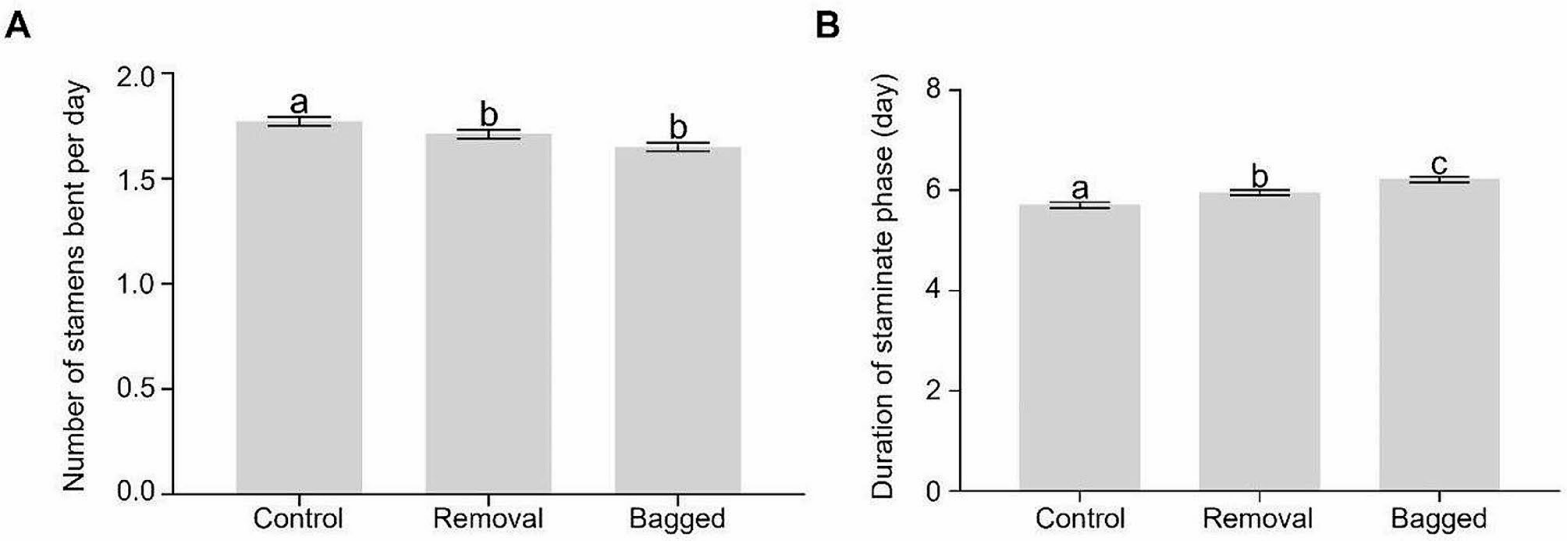
Number of stamens bent per day (**A**) and duration of staminate phase (day) (**B**) in flowers of *Saxifraga candelabrum* among different treatments of pollinator visits

Bagged flowers, which completely excluded pollinator visitation, appeared to have the fewest stamens bent daily, while the treatment of pollen removal imitating one to two pollinator visits had slightly more stamens bent (although not statistically significant, Fig. 3A). Open pollination had the highest number of stamens bent (*F*_2,444_ = 14.3, *P* < 0.0001; Fig. 3A). Such a difference in the rate of stamen movement continued to affect the duration of staminate phase, and the pattern was exactly the opposite. With the increase of pollinator visits (from bagged to removal to control), the duration of staminate phase decreased remarkably (*F*_2,409_ = 25.5, *P* < 0.0001; Fig. 3B).

### Seasonal changes and schedule of pollen presentation

**Fig. 4:**
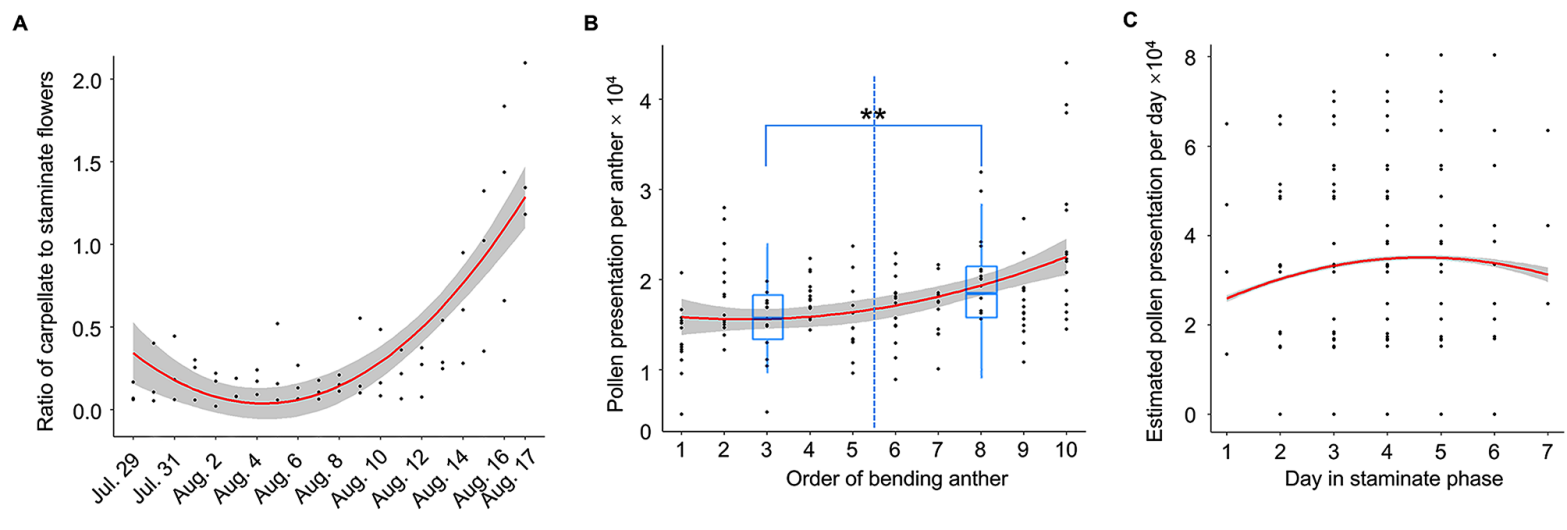
The ratio of carpellate- to staminate-phase flowers during the peak of anthesis (observation period from Jul. 29 to Aug. 17) (**A**); the pollen presentation per anther with the order of bending in *Saxifraga candelabrum* (**B**) and estimated pollen presentation per day with the progress of staminate phase of flowers of *Saxifraga candelabrum* (**C**). The blue boxplots represent the pollen presentation per anther for the antesepalous (order 1–5) and the antepetalous (order 6–10) stamens. ** indicates *P* < 0.01. The gray area represents the 95% confidence interval for regression lines

There was a significant quadratic regression of the population flower sex ratio on dates (sex ratio = 0.008×date^2^ – 0.113×date + 0.45, R^2^ = 0.67, *F*_2,17_ = 57.45, *P* < 0.0001; Fig. 4A). The population was male-dominated for the first week of anthesis, before it steadily shifted toward femaleness. However, the rate of stamen movement was not different between early-season flowers (1.72 ± 0.05 stamens per day) and mid-season ones (1.70 ± 0.05; *F*_1,662_ = 0.1, *P* = 0.77).

At within-flower level, rather than an equal packaging scheme, pollen production per anther had an increasing trend with the order of bending in flowers of *S. candelabrum* (pollen = 121.93×order^2^ – 598.51×order + 16345.47, R^2^ = 0.17, *F*_1,144_ = 30.91, *P* < 0.0001; Fig. 4B). The anther that moved last in a flower had 84.1% more pollen than the first one. The successive increase may be partly due to developmental differences since when the comparison was made between the antesepalous and antepetalous groups, they indeed differed in pollen quantity (t = 3.88, *P* = 0.0002; boxplots in Fig. 4B). However, when the group difference was controlled, the bending order still showed a significant positive effect on pollen quantity for each group (*F*_1,143_ = 11.72, *P* = 0.0008), indicating that bending order played a role. Furthermore, the daily number of pollen presentation also showed a quadratic pattern with the progress of staminate phase (daily pollen presentation = – 717.85×day^2^ + 6120.416×day + 20732.073, *F*_1,7140.5_ = 157.47, *P* < 0.0001; Fig. 4C). The trend implied that pollen presentation stably increased for the first 5 days, and the slight decrease for day 6 and 7 was probably due to limited number of stamens left, part of which also displayed wider errors (Fig. 4C).

## Discussion

Stamens of *Saxifraga candelabrum* exhibited a successive movement, a feature also found in the Parnassiaceae, Rutaceae, Tropaeolaceae, and Loasaceae [15]. The adaptive significance of such stamen movements has been proposed to reduce male-female and male-male interference [17], increase pollen deposition [16], promote pollen export [43], and achieve reproductive assurance [18]. In flowers of *S. candelabrum*, the staminate and carpellate phases are distinctively separated, which precludes male-female interactions and reproductive assurance via selfing. Moreover, the anther sacs are generally detached from stamens when they move back to original positions (Fig. 1). It appears that the movement has no further role in minimizing anther-anther interference. Hence, we suppose that the stamen movement in *S. candelabrum* function in enhancing pollen export, especially under alpine conditions where both weather and pollinator activities are highly unpredictable.

Results showed that there were considerably more stamens bent in flowers of *S. candelabrum* on sunny days than on cloudy or rainy days. According to Henning and Weigend [44], the autonomous successive movements of eight species of Loasaceae were found to be positively influenced by temperature and light, and the stamens would come to a virtual standstill in the dark and at low temperatures (12℃). It suggests that the stamen development can be externally cued by temperature and light. In addition, there were severe negative effects of rain on pollen viability and effective pollination [45–47], indicating that increased humidity in overcast and rainy conditions might have also played a role in slowing down the movement of stamen in *S. candelabrum*. Besides abiotic factors, pollinator visits also displayed coupled changes with weather conditions. Therefore, it was possible that pollinator activity can accelerate stamen movement. Indeed, the successive movement of *Ruta graveolens* L. speeded up when visited by a large number of insects in a short time [18]. *Nasa dyeri* (Urb. & Gilg) Weigend ssp. *australis* Dostert and Weigend also shortened the duration of the staminate phase from 3.4 days when there was no pollinator visit to 2.7 days when there were three pollinator visits per day [48]. However, to what extent the cascading development of stamens can be directly regulated by pollinator activities in Saxifragaceae lacks empirical investigation.

By employing artificial manipulations of pollen removal and bagging, we showed that the number of stamens bent daily under open pollination was significantly higher than that of removal (3.40% increase) and bagged (7.27% increase). Accordingly, flowers with open pollination had a shortened staminate phase by 4.19% than the pollen removal treatment and by 8.20% than bagging. This demonstrates that pollinator activities alone can influence the rate of successive movement in *S. candelabrum*. Such a flexible response likely reflects an adaption to highly variable pollination service, slowing down the movement and thus saving pollen when pollinators are scarce, whereas speeding up the movement and donating pollen when visitation rates rise. It is worth noting that the effect is independent of abiotic factors, as all treatments were under the same weather conditions. Although pollinator visits have been found to influence developmental schedules of stamens [49] and floral longevity [50, 51], our study provides solid evidence of effects of pollinator visits on successive stamen movements.

Protandry in *S. saxifraga* results in a seasonal shift in sex ratio from male-dominated to almost pure female flower population [28, 52]. Contrary to our prediction [29], mid-season flowers did not speed up stamen behavior compared to early-season ones. However, increasing trends were obvious when we looked at both pollen packaging and daily pollen presentation (Fig. 4B & C). Albeit ontogenetic factors seem to affect pollen production as the antesepalous stamens matured early and produced fewer pollen than the antepetalous ones, the bending order still showed a clear impact in that pollen amount per anther increased successively. It seems that the pattern in pollen presentation was largely driven by growing pollen production per anther, because the other deterministic component of pollen presentation, number of bent stamens per day, was strongly influenced by fluctuations in weather and pollinator visits (see Figs. 2 and 3), which is unpredictable. Therefore, the incremental pollen packaging may be a more realistic and steady response to negative frequency-dependent selection shaped by the skewed sex ratio [53]. Later displayed anthers in general face more mating opportunities as there were more ovules (Fig. 4A). One may wonder that the within-flower pollen packaging strategy cannot scale up to match population seasonal changes. Nonetheless, compared to the compact flowering period of one month for alpine herbs like *S. candelabrum*, a week-long staminate phase constitutes nearly one fourth of the season. Such a span is wide enough to experience population-level change in sex ratio and hence may allow response to selection. Therefore, while the rate of stamen movement is more responsive to local ecological perturbations, the incremental pollen packaging is likely an adaptation to increase male reproduction under the seasonal regularity of population sex ratio.

The fitness consequence of successive stamen movement in *S. candelabrum* should lie in promoting pollen export and accomplishing male reproductive success. Such a movement, in essence, staggers low doses of pollen presentation presented across several days of pollination episodes. On the one hand, the small packages of pollen at any given moment would not lead to massive pollen wastage either caused by rain wash-off [54] or insects of low pollination efficiency (e.g. flies, beetles; [55, 56]), both of which are common phenomena in its natural habitat. On the other hand, given the highly variable weather plants of *S. candelabrum* experience [57], successive stamen movement allows an extension of pollen exposure duration, thus facilitating pollen export through the accumulation of visits paid by various groups of pollinators. Similar to the results of *Ruta graveolens* L., *Parnassia palustris* and *Parnassia wightiana* Wall. ex Wight & Arn. [17, 18, 43], all with successive stamen movement, our experiments also found the reproduction of *S. candelabrum* was pollen-limited, which highlights strong selection pressure to maximize pollen transfer. To consolidate the adaptiveness of successive stamen movement, it is ideal to compare pollen transfer and seed sired between flowers with such behaviors and those without. Unfortunately, manipulative experiments are almost impossible to conduct because we cannot make all stamens mature and move at once or delay the developmental schedule of certain ones. Nonetheless, a few studies have tried to fix mature stamens with threads or tapes from bending to the center or moving away in *Parnassia* spp. [17, 43] and *Ruta graveolens* [18]. Their results showed that manipulations had either reduced pollen visitation or pollen transfer efficiency, resulting in fewer seed set. Yet, the focus of the above studies is primarily on anther-anther interference, which is unlikely the selective force in *S. candelabrum* as previously elaborated. Hence, to gain a deeper understanding of stamen behavior in *S. candelabrum*, we need more studies on the quantification of pollen transfer and reproduction outcomes.

## Conclusion

Our study not only adds a new family, Saxifragaceae, to the list of plants that exhibit successive stamen movement, but also demonstrates that such spontaneous behavior can be flexibly regulated by abiotic and biotic factors to improve pollen donation. In addition, our work reveals that incremental pollen packaging and presentation appear to be an adaptation to seasonal increase in ovule availability. Very few studies have examined variations in pollen production among anthers. We, however, provide solid evidence of changes in pollen packaging and presentation of *S. candelabrum*. Considering the prevalence of dichogamy in angiosperms [58], which can lead to regular patterns in population sex ratio, reproductive strategies involving alterable gamete allocation may have been underexplored.

### Electronic supplementary material

Below is the link to the electronic supplementary material.

Supplementary Material 1

Supplementary Material 2

## Data Availability

No datasets were generated or analysed during the current study.
