# Supplementary figures and images for: Successive stamen movement in Saxifraga candelabrum is responsive to weather and pollinator visits
Source: Mov Ecol. 2024 Jun 8;12:43. doi: 10.1186/s40462-024-00483-9 (PMC11162003; doi:10.1186/s40462-024-00483-9)

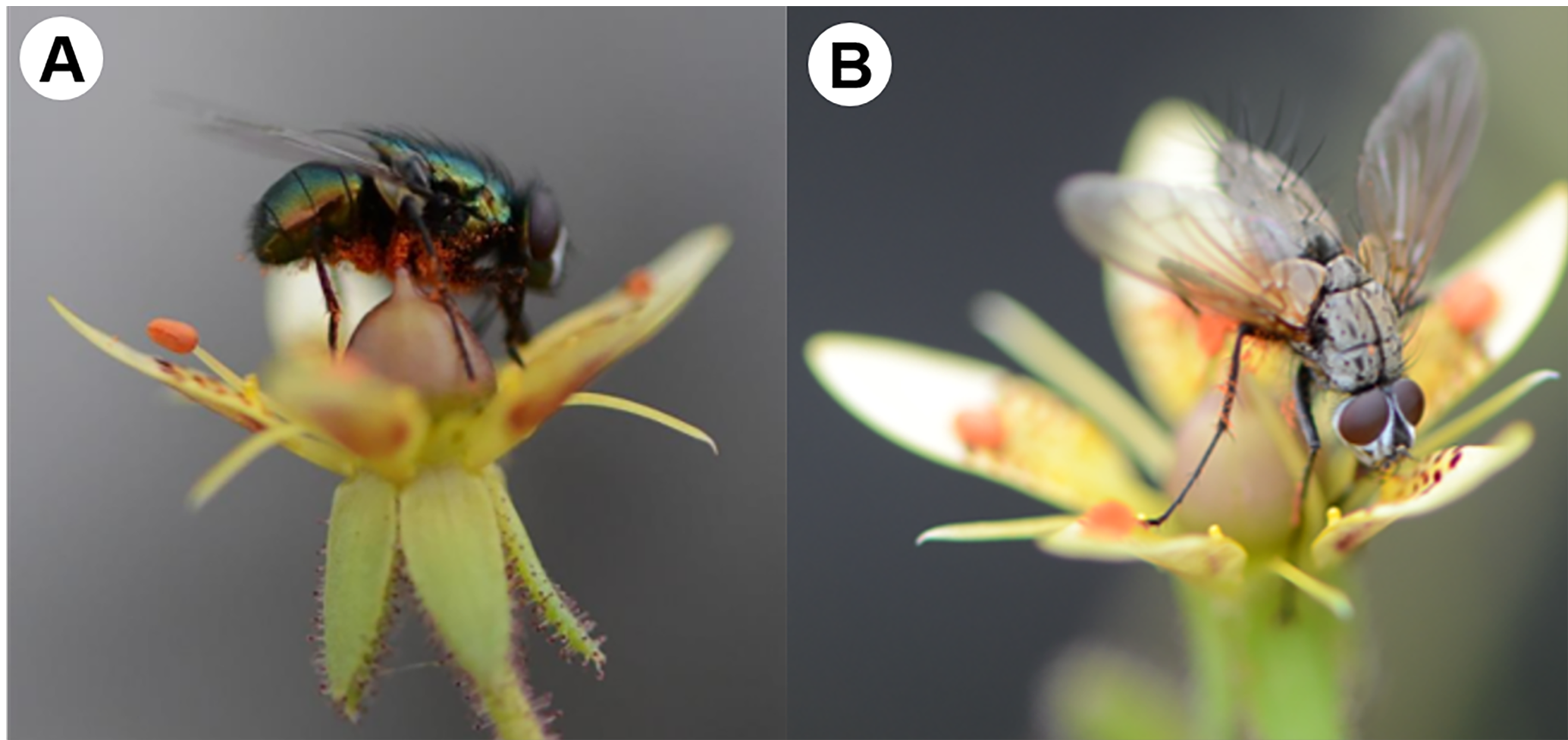

Supplement: Supplementary file 1 — Supplementary Material 1 [file 40462_2024_483_MOESM1_ESM.tif]

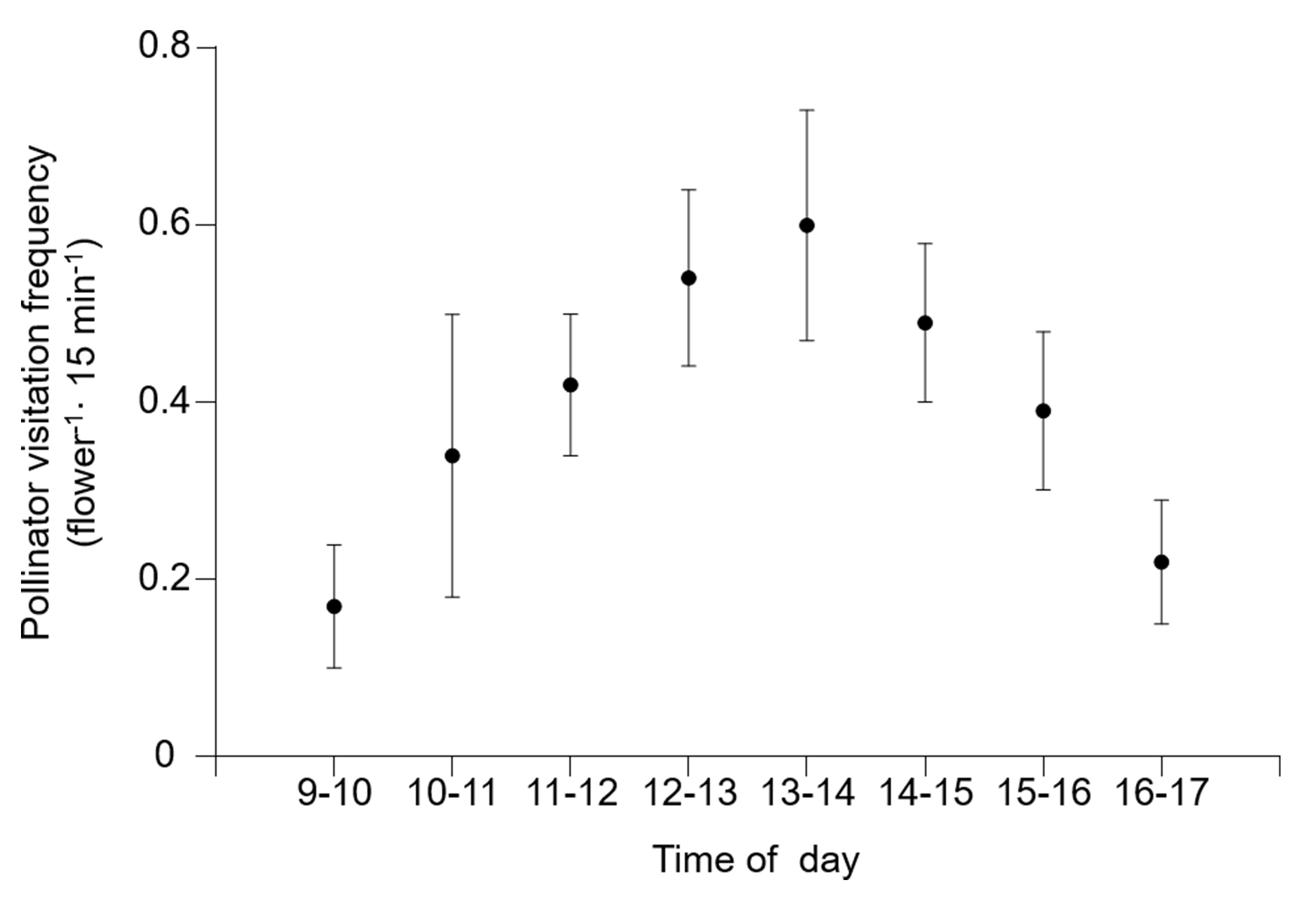

Supplement: Supplementary file 2 — Supplementary Material 2 [file 40462_2024_483_MOESM2_ESM.tif]
